# Hyperthermia is a predictor of high mortality in patients with sepsis

**DOI:** 10.1186/s13054-020-03263-0

**Published:** 2020-09-03

**Authors:** Yanfei Shen, Yangfang Lou, Shiping Zhu

**Affiliations:** 1 Department of Intensive Care, Zhejiang Hospital, 1220#, Gudun-Road, Hangzhou, Zhejiang China; 2 Department of Respiratory Medicine, Hangzhou Hospital of Traditional Chinese Medicine, No. 453, Tiyuchang Road, Hangzhou, 310000 Zhejiang China

To the Editor,

In a recent study, Shimazui et al. [1] reported that body temperature (BT) on ICU admission exhibited different predictive values in elderly and non-elderly patients with sepsis, and only hypothermia (BT < 36.0 °C) was associated with increased mortality in non-elderly patients while hyperthermia (BT > 38.3 °C) was not. A few issues should be noted.

First, the grouping method may underestimate the impact of hyperthermia. In the current study, the whole cohort was divided into the hyperthermia and non-hyperthermia groups, using a cutoff value of BT at 38.3 °C. One limitation is that under this grouping method, both hypothermia and normothermia were classified as non-hyperthermia. Thus, the comparison between the hyperthermia and non-hyperthermia groups could be susceptible to the proportion of patients with hypothermia. For instance, in two hypothetical cohorts (cohort 1: hypothermia *n* = 80, normothermia *n* = 20, hyperthermia *n* = 100 vs. cohort 2: hypothermia *n* = 20, normothermia *n* = 80, hyperthermia *n* = 100), the comparison of mortality between the hyperthermia and non-hyperthermia groups could be quite different in these two cohorts, as the non-hyperthermia group in cohort 1 (high proportion of hypothermia patients) may have high mortality. In addition, several studies [2] also reported that in sepsis, hyperthermia (T_max_) was also a significant risk for high mortality. Furthermore, one randomized controlled trial (RCT) found that fever control using external cooling to maintain BT between 36.5 and 37.0 °C significantly reduced mortality in septic shock [3]. For validation, we explored the association between BT and mortality in another cohort from MIMIC-III database (Fig. 1). A total of 4201 adult patients with sepsis were included. Consistent with the current study, different associations between BT and mortality were also found in old (≥ 75) and young (< 75) patients. However, in patients with age < 75, both hypothermia and hyperthermia exhibited increased trends of in-hospital mortality (Fig. 1 black bars).

**Fig. 1:**
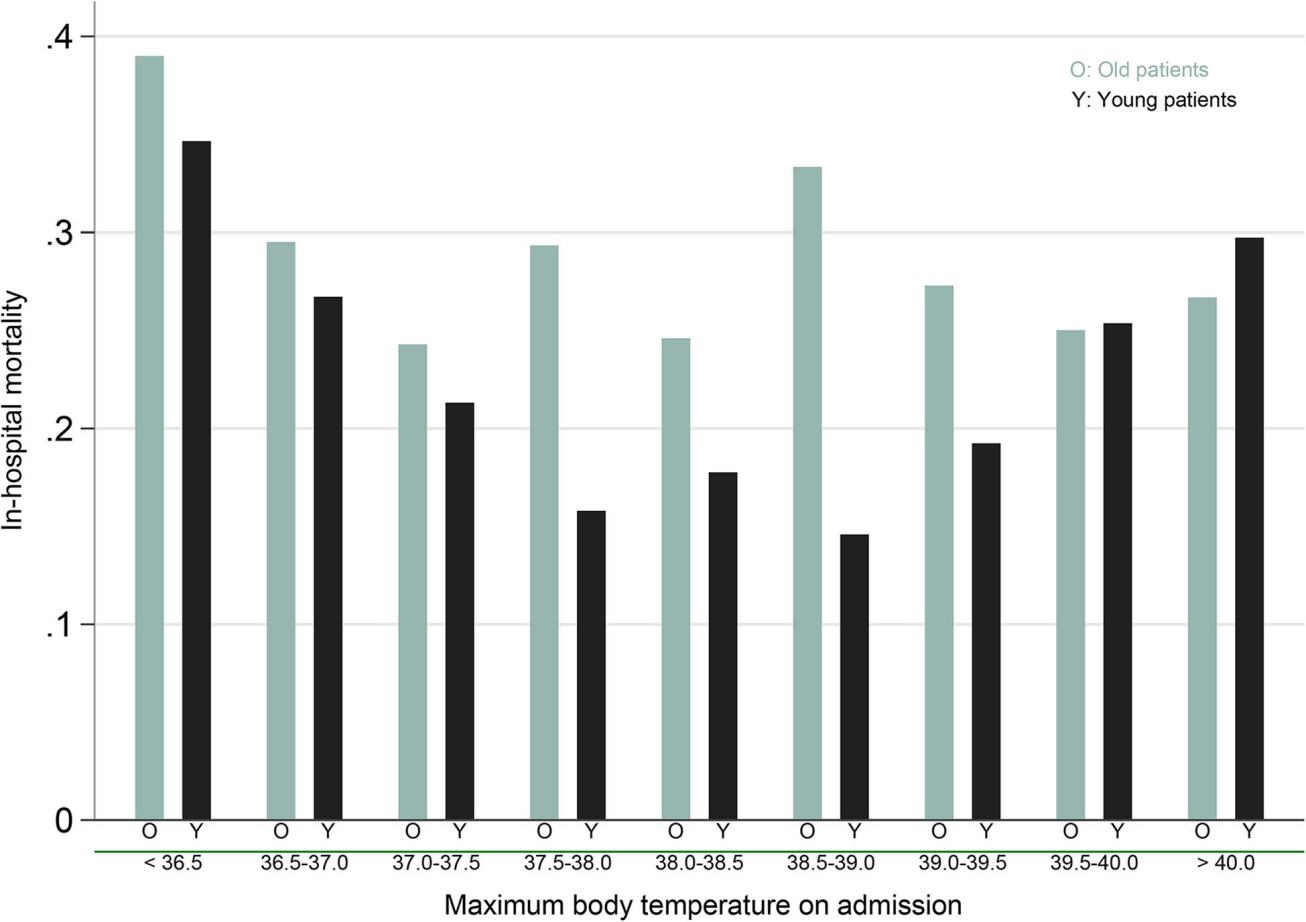
Association between body temperature and in-hospital mortality in old (≥ 75 years old) and young (< 75 years old) patients with sepsis

Second, the author mentioned that the impact of hypothermia duration on mortality remained unclear. Noteworthy, in a median analysis of previous RCT [3], Schortgen et al. [4] found that 73% of the impact of external cooling on mortality was mediated by the duration of BT < 38.4 °C. Thus, focusing on a single BT record may increase the bias risk. Temperature load (TL) [5] may be a method to this limitation, defined as the sum of BT above/below the targeted temperature level multiplied by the duration (hours). For instance, the TL of hyperthermia (> 38.3 °C) within 72 h should be calculated as follows—step 1: $\overline{t_i}=\frac{t_i+{\mathrm{t}}_{\mathrm{i}+1}}{2}-38.3$; step 2: $\mathrm{TL}={\sum}_{i=1}^{72}\overline{t_i}\times 1\ \mathrm{hour}$.

## Data Availability

Not applicable.
